# Spatial–temporal graph convolutional network for Alzheimer classification based on brain functional connectivity imaging of electroencephalogram

**DOI:** 10.1002/hbm.25994

**Published:** 2022-06-25

**Authors:** Xiaocai Shan, Jun Cao, Shoudong Huo, Liangyu Chen, Ptolemaios Georgios Sarrigiannis, Yifan Zhao

**Affiliations:** ^1^ Institute of Geology and Geophysics Chinese Academy of Sciences Beijing China; ^2^ School of Aerospace, Transport and Manufacturing Cranfield University Cranfield UK; ^3^ Department of Neurosurgery Shengjing Hospital of China Medical University Shenyang China; ^4^ Royal Devon and Exeter NHS Foundation Trust Exeter UK

**Keywords:** artificial intelligence, brain association, electroencephalogram, graph convolutional neural network, machine learning

## Abstract

Functional connectivity of the human brain, representing statistical dependence of information flow between cortical regions, significantly contributes to the study of the intrinsic brain network and its functional mechanism. To fully explore its potential in the early diagnosis of Alzheimer's disease (AD) using electroencephalogram (EEG) recordings, this article introduces a novel dynamical spatial–temporal graph convolutional neural network (ST‐GCN) for better classification performance. Different from existing studies that are based on either topological brain function characteristics or temporal features of EEG, the proposed ST‐GCN considers both the adjacency matrix of functional connectivity from multiple EEG channels and corresponding dynamics of signal EEG channel simultaneously. Different from the traditional graph convolutional neural networks, the proposed ST‐GCN makes full use of the constrained spatial topology of functional connectivity and the discriminative dynamic temporal information represented by the 1D convolution. We conducted extensive experiments on the clinical EEG data set of AD patients and Healthy Controls. The results demonstrate that the proposed method achieves better classification performance (92.3%) than the state‐of‐the‐art methods. This approach can not only help diagnose AD but also better understand the effect of normal ageing on brain network characteristics before we can accurately diagnose the condition based on resting‐state EEG.

## Graphical abstract

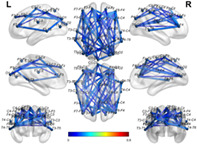

## 1 INTRODUCTION

Alzheimer's disease (AD) is the most common form of dementia, resulting in the loss of memory and cognitive impairments. Most commonly it occurs among elderly people over the age of 65, but it may also occur earlier (Brookmeyer et al., 1998, 2007). According to the World Health Organization, more than 55 million people are currently diagnosed with some type of dementia (World Health Organization, 2021). Due to increasing global ageing, the technologies to diagnose AD more effectively and accurately are highly demanded. Numerous studies have shown that synaptic dysfunction is an early feature of AD and that decreased synaptic density in the neocortex and limbic regions could account for AD‐associated disturbances in brain function (Masliah et al., 2001; Scheff et al., 2006, 2007; Terry et al., 1991). Being more extensive than the corresponding neuronal loss when analysed in the same brain regions, synaptic dysfunction has been proved to be the best neuropathological correlate of cognitive impairment in AD patients (Davies et al., 1987; DeKosky & Scheff, 1990; Scheff et al., 2007; Terry et al., 1991). The pathological progression of AD leads to cortical disconnections and manifests as functional connectivity alterations (Nobukawa et al., 2020). Electroencephalography (EEG) is a noninvasive diagnostic method for studying the bioelectrical function alterations and degeneration of the brain. Consisting of scalp electric potential differences, EEG is one of the first measurements that directly reflect the functioning of synapses in real‐time (Jelic, 2005; Michel et al., 2009). In contrast to functional MRI or PET which detect indirect metabolic signals, EEG offers several additional attractions: noninvasiveness, high time‐resolution, wide availability, low cost, and direct access to neuronal signalling (Michel et al., 2009; Smailovic et al., 2018).

For the diagnosis of AD based on EEG, there are two main development areas in recent years. The first area is statistically topological brain function characteristics. Finding statistical biomarkers of AD is based on analysing graph topographic or dynamic patterns. For graph topography, Jalili (2017) constructed functional brain networks and relevant graph theory metrics based on EEG for discriminating AD from Healthy Controls (HC). Similarly, Tylová et al. (2018) proposed the permutation entropy for measuring the chaotic behaviour of EEG and observed a statistically significant decrease in permutation entropy at all channels of AD. Fan et al. (2018) employed multiscale entropy as the biomarker to characterize the nonlinear complexity at multiple temporal scales to capture the topographic pattern of AD. For dynamic patterns, Zhao et al. (2019) proposed a method to measure nonlinear dynamics of functional connectivity for distinguishing between AD and HC, and Tait et al. (2020) developed a biomarker by combining microstate transitioning complexity and the spectral measure. The second area is data‐driven machine learning or deep learning methods with various input features for the AD classification. Researchers have proven that SVM (Nobukawa et al., 2020; Song et al., 2018; Tavares et al., 2019; Trambaiolli et al., 2017), fuzzy model (Yu et al., 2019), KNN (Safi & Safi, 2021), bagged trees (Oltu et al., 2021), and artificial neural network (ANN) (Ieracitano et al., 2020; Rodrigues et al., 2021; Triggiani et al., 2017) can help test the validity of the input topographic or dynamic patterns or features, such as multiscale entropy vector (Song et al., 2018), functional connectivity (Nobukawa et al., 2020; Song et al., 2018; Yu et al., 2019), cepstral distances (Rodrigues et al., 2021), Hjorth parameters (Safi & Safi, 2021), coherence (Oltu et al., 2021), bispectrum (Ieracitano et al., 2020), power spectral density (Oltu et al., 2021; Tavares et al., 2019), frequency bands (Trambaiolli et al., 2017; Triggiani et al., 2017), and wavelet transform (Ieracitano et al., 2020; Oltu et al., 2021).

Among these machine learning methods, it has been proven that deep learning has exceptional performance in terms of the accuracy of classification. Convolutional neural networks (CNN), a leading deep learning structure for data on Euclidean space, outperform the above machine learning algorithms in classification accuracy (Craik et al., 2019). Based on the fast Fourier transform for extracting spectral features of EEG for AD diagnosis, Bi and Wang (2019) developed a discriminative convolutional Boltzmann machine; Ieracitano et al. (2019) and Deepthi et al. (2020) proposed a CNN model, respectively. Based on combining latent factors output by the encoder part of variational auto‐encoder of EEG, Li, Wang, et al. (2021) extracted characteristics of AD. Based on the time‐frequency analysis using CWT, Huggins et al. (2021) proposed an AlexNet model for the classification of AD, mild cognitive impairment subjects, and HC. With a connections matrix from EEG, Alves et al. (2021) presented a CNN for classifying AD and schizophrenia. The above CNN applications on AD classification (Bi & Wang, 2019; Deepthi et al., 2020; Huggins et al., 2021; Ieracitano et al., 2019; Li, Wang, et al., 2021) focus more on learning the locally and continuously changed multiscaled features on the Euclidean space from the EEG signals, neglecting the functional connectivity features. Although Alves et al. (2021) used the connection of EEG channels as the CNN input, it neglected the temporal EEG channels features and the input connections topology feature cannot be modelled effectively due to the arranged order of EEG channels. The key convolutional filters on CNN structures in the above applications cannot fully mine the multiscale topological interactive information of EEG channels.

Considering the complexity of EEG signals in the spatial and temporal domain, how to extract more abstract geometric features for better generalisation using the deep learning methods remains tremendously troubling. The structure–function connectivity network of EEG is non‐Euclidean data because the channels are discrete and discontinuous in the spatial domain. Each EEG channel can be considered as a node and there is a cross‐channel interaction between nodes. Instead, geometric graph‐based deep learning methods would provide a more suitable way to learn the cross‐channel topologically associated features of EEG. Building neural networks under the graph theory, graph convolutional neural networks (GCNs) have been developed specifically to handle highly multirelational graph data by jointly leveraging node‐specific sequential features and cross‐nodes topologically associative features in the graph domain (Gallicchio & Micheli, 2010; Gori et al., 2005; Scarselli et al., 2008; Sperduti & Starita, 1997). In recent 2 years, GCNs have been applied in the diagnoses of various brain disorders, such as children's ASD evaluation (Zhang et al., 2021), detection of epileptic (Zeng et al., 2020; Zhao et al., 2021), seizure prediction (Li, Liu, et al., 2021), and epilepsy classification (Chen et al., 2020). As far as we are concerned, there are no AD diagnostic approaches based on GCN‐related models.

To enable this application, an adjacency matrix, representing the topological association between different EEG channels, must be constructed as the key input of GCN. EEG functional networks are widely used in cognitive neuroscience, for example, decision making (Si et al., 2019), emotion recognition (Li, Liu, et al., 2019), and Schizophrenia research (Li, Wang, et al., 2019). Traditional statistical functional correlation measures include Pearson correlation (PC) (Chen et al., 2020; Zhao et al., 2021), Tanh nonlinearity (Li, Liu, et al., 2021), average correlation coefficients (Zeng et al., 2020), and covariance (Zhang et al., 2021), which cannot fully represent the complex brain connectivity. Many advanced methods to measure functional connectivity (FC) have been developed, such as phase locking values (PLV) and phase lag index (PLI) in the time domain (Franciotti et al., 2019; Mormann et al., 2000; Van Mierlo et al., 2014), magnitude squared coherence (MSC) and imaginary part of coherence (IPC) in the frequency domain (Al‐Ezzi et al., 2021; Babiloni et al., 2005; Van Diessen et al., 2015; Wendling et al., 2009), and wavelet coherence (WC) in the time–frequency domain (Franciotti et al., 2019). These values can measure the degree of synchronisation between different brain regions and alterations in complex behaviours produced by the interaction among widespread brain regions (Babiloni et al., 2005; Sakkalis, 2011; Tafreshi et al., 2019; Van Mierlo et al., 2014), which have been proved important for AD classification using the statistical (Jalili, 2017; Zhao et al., 2019), and machine learning methods (Nobukawa et al., 2020; Song et al., 2018; Yu et al., 2019). However, the research on the combination of FC and GCN is limited, especially for AD‐related research. Using these efficient FCs to construct the input adjacency matrix of GCN may promisingly provide more insightful information for the brain function interaction and lead to a higher classification accuracy of brain‐related disorders.

In this article, a novel spatial–temporal GCN (ST‐GCN) is proposed to classify AD from HC, benefiting from the adjacency matrix constructed by a variety of FC measures and the raw EEG recordings. We tested six adjacency matrices based on PC, MSC, IPC, WC, PLV, and PLI using EEG recordings from patients with AD and HCs. ST‐GCN can jointly leverage the cross‐channel topological connectivity features and channel‐specific temporal features. To the best of the authors' knowledge, this is the first attempt for GCN to distinguish between AD and HC based on EEG recordings.

## 2 METHODS

### 2.1 Spatial–temporal graph convolutional network

In 1997, Sperduti and Starita first adopted neural networks to direct acyclic graphs (Sperduti & Starita, 1997), which motivated the early studies on GCNs (Gallicchio & Micheli, 2010; Gori et al., 2005; Scarselli et al., 2008). Currently, there are two basic approaches to generalising convolutions to structure graph data forms: spatial‐based and spectral‐based GNNs. Spatial‐based GNNs define graph convolutions by rearranging vertices into certain grid forms which can be processed by normal convolutional operations (Niepert et al., 2016; Yu et al., 2017). Bruna et al. (2013) presented the first prominent spectral‐based GCNs by applying convolutions in spectral domains with graph Fourier transforms. Since then, there have been increasing improvements, approximations, and extensions on spectral‐based GNNs (Defferrard et al., 2016; Henaff et al., 2015; Kipf & Welling, 2016; Levie et al., 2018) to reduce the computational complexity from $O\left(n^{2}\right)$ to $O\left(n\right)$ (Defferrard et al., 2016; Kipf & Welling, 2016). Visually, a graph convolution can handle the complexity of graph data by generalising a 2D convolution, motivated by the successful applications of CNNs in Euclidean space (Wu et al., 2020). Being considered as special graph data, each pixel of an image can be taken as a node whose neighbours are determined by a filter and a 2D convolution takes the weighted average of adjacent pixel values of each node. Similarly, graph convolutions can be performed by taking the weighted average of a node's neighbourhood information, which is unordered and variable in size, and different from images.

**FIGURE 1:**
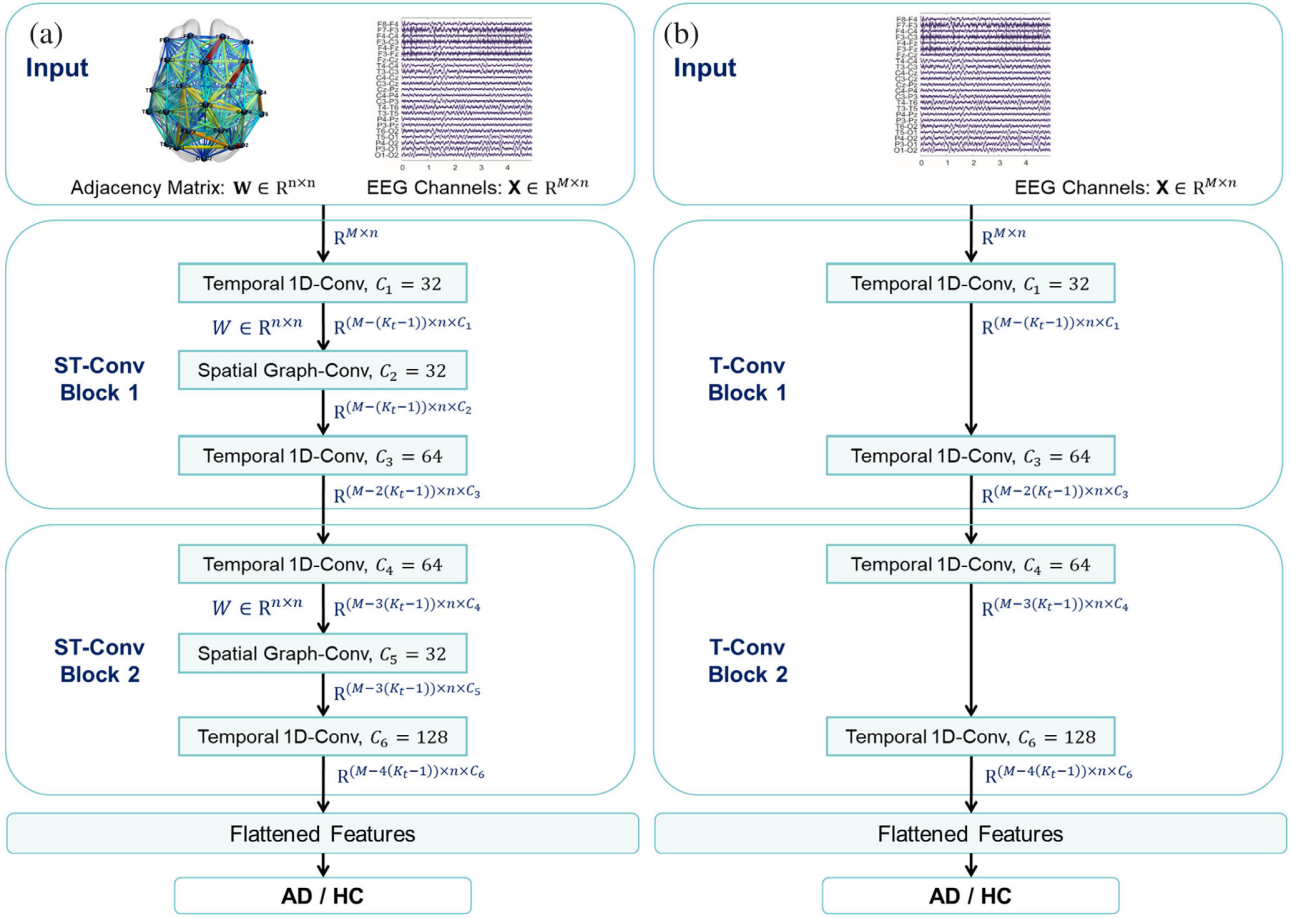
The flowchart of (a) the proposed ST‐GCN framework and (b) the T‐CNN framework for comparison. X is sized M*N (*M* = 25, representing the length of the mini‐epoch channel; *N* = 23, representing the 23 channels) and the input W is sized N*N. The (*i*,*j*)th entry of the adjacency matrix W denotes spatial coupling correlation strength between the *i*th and *j*th of all the 23 different channels, the detailed calculation of which is presented in section 2.2. $K_{t}$ is the size of the temporal 1D‐Conv filter, set as 3 here. $C_{l}\;\left(l=1,2,\ldots ,6\right)$ is the number of filters in each layer. The estimated computational complexity of ST‐GCN and T‐CNN are 54M and 52M, respectively

As shown in Figure 1a, the proposed architecture of ST‐GCN is composed of two spatial–temporal convolutional blocks (ST‐Conv Blocks), each of which is formed with one spatial graph convolution layer (Spatial Graph‐Conv) and two sequential convolution layers (Temporal 1D‐Conv). ST‐Conv block can be stacked based on the complexity of specific cases. Layer normalisation is utilised within every ST‐Conv Block to prevent overfitting. The EEG channels *X* with the adjacency matrix *W* are uniformly processed by ST‐Conv Blocks to explore spatial and temporal dependence coherently. A flattened layer integrates comprehensive features to generate the final AD/HC classification.

For comparison, we designed a structure of a classical temporal convolutional neural network (T‐CNN) shown in Figure 1b, inspired by some related works (Deepthi et al., 2020; Huggins et al., 2021; Li, Wang, et al., 2021). The difference between these two model structures is that T‐CNN only has EEG channels *X* at the input without the adjacency matrix *W*, and no spatial graph convolution unit is used in the feature layer in the middle of the T‐CNN. Other aspects of T‐CNN are the same as ST‐GCN. For brevity, we illustrate the structural details of each part of ST‐GCN in the following.

#### 2.1.1 Spatial Graph‐Conv

Based on the concept of a spectral graph convolution, we introduce the notion of a graph convolution operator “*G,” multiplying a signal $x\in R^{n}$ in the spatial space with a kernel Θ,

(1) $$\Theta *G\;x=\Theta \left(L\right)x=\Theta \left(\mathrm{U\Lambda}U^{T}\right)x=\mathrm{U\Theta}\left(\Lambda \right)U^{T}x,$$

where the graph Fourier basis $U\in R^{n\times n}$ is a matrix of eigenvectors of the normalised graph Laplacian $L=I_{n}-D^{-\frac{1}{2}}WD^{-\frac{1}{2}}=\mathrm{U\Lambda}U^{T}\in R^{n\times n}$ ($I_{n}$ is an identity matrix, $D\in R^{n\times n}$ is the diagonal degree matrix with $D_{\mathrm{ii}}=\sum_{j}W_{\mathrm{ij}}$, $\Lambda \in R^{n\times n}$ is the diagonal matrix of eigenvalues of L, and filter $\Theta \left(\Lambda \right)$ is a diagonal matrix). A graph signal x is filtered by a kernel Θ with multiplication between Θ and the graph Fourier transform $U^{T}x$ (Shuman et al., 2013).

We utilise Chebyshev polynomials and first‐order approximations (Kipf & Welling, 2016) here to reduce expensive computations of kernel Θ in Equation (1) due to its $O\left(n^{2}\right)$ complex multiplications. Kernel Θ can be restricted to a polynomial of Λ as $\Theta \left(\Lambda \right)=\sum_{k=0}^{K-1}\theta_{k}\Lambda^{k}$, where $\theta \in R^{K}$ is a vector of polynomial coefficients and *K* is the kernel size determining the maximum radius of the convolution from central nodes. Chebyshev polynomial $T_{k}\left(x\right)$ is traditionally used to approximate kernels as a truncated expansion of order K−1 as $\Theta \left(\Lambda \right)\approx \sum_{k=0}^{K-1}\theta_{k}T_{k}\left(\tilde{\Lambda }\right)$ by rescaled $\tilde{\Lambda }=2\Lambda /\lambda_{\max}-I_{n}$, where $\lambda_{\max}$ denotes the largest eigenvalue of *L* (Hammond et al., 2011). Then the graph convolution in Equation (1) can be rewritten as

(2) $$\Theta *G\;x=\Theta \left(L\right)x\approx \sum_{k=0}^{K-1}\theta_{k}T_{k}\left(\tilde{L}\right)x,$$

where $T_{k}\left(\tilde{L}\right)\in R^{n\times n}$ is the Chebyshev polynomial of order k evaluated at the scaled Laplacian $\tilde{L}=2L/\lambda_{\max}-I_{n}$. By recursively computing *K*‐localised convolutions through a polynomial approximation, the computational cost of Equation (1) can be reduced to $O\left(K\left|\varepsilon \right|\right)$ as Equation (2). By stacking multiple localised graph convolutional layers with a first‐order approximation of graph Laplacian, a layer‐wise linear formulation can be defined (Kipf & Welling, 2016). Further assumption $\lambda_{\max}\approx 2$ can be made, due to the scaling and normalisation in neural networks. Thus, Equation (2) can be simplified to

(3) $$\Theta *G\;x\approx \theta_{0}x+\theta_{1}\left(\frac{2}{\lambda_{\max}}L-I_{n}\right)x\approx \theta_{0}x-\theta_{1}\left(D^{-\frac{1}{2}}WD^{-\frac{1}{2}}\right)x，$$

where $\theta_{0}\;\text{and}\;\theta_{1}$ are two shared parameters of the kernel. $\theta_{0}$ and $\theta_{1}$ are replaced by a single parameter θ by letting $\theta_{0}=-\theta_{1}=\theta$ to constrain parameters and stabilise numerical performances. By renormalising *W* and *D* with $\tilde{W}=W+I_{n}$ and ${\tilde{D}}_{\mathrm{ii}}=\sum_{j}{\tilde{W}}_{\mathrm{ij}}$ separately, graph convolution can be expressed as

(4) $$\Theta *G\;x=\theta \left(I_{n}+D^{-\frac{1}{2}}\;W\;D^{-\frac{1}{2}}\right)x=\theta \left({\tilde{D}}^{-\frac{1}{2}}\;\tilde{W\;}{\tilde{D}}^{-\frac{1}{2}}\right)x.$$

The graph convolution operator “*G” defined on $x\in R^{n}$ can be extended to multidimensional tensors. For a signal with $C_{i}$ channels $X\in R^{n\times C_{i}}$, the graph convolution can be generalised to

(5) $$y_{j}=\sum_{i=1}^{C_{i}}\Theta_{i,j}\left(L\right)x_{i}\in R^{n},1\leq j\leq C_{0},$$

with the $C_{i}\times C_{0}$ vectors of Chebyshev coefficients $\Theta_{i,j}\in R^{K}$ ($y_{j}$ is the output after graph convolution, $C_{i}$ and $C_{0}$ are the sizes of input and output of the feature maps, respectively). A graph convolution for 2D variables is denoted as “Θ*GX” with $\Theta \in R^{K\times C_{i}\times C_{0}}$. The input of ST‐GCN is composed of *M* frames of EEG channels graph as shown in Figure 1a. Each frame $X_{t}$ can be regarded as a matrix whose column i is a $C_{i}$‐dimensional value of $X_{t}$ at the *i*th node in graph $G_{t}$, as $X\in R^{n\times C_{i}}$ (in this case, $C_{i}=1$). For each time step *t* of *M*, the equal graph convolution operation with the same kernel Θ is imposed on $X_{t}\in R^{n\times C_{i}}$ in parallel. Thus, the graph convolution can be generalised to 3D variables, noted as “Θ*Gχ” with $\chi \in R^{M\times n\times C_{i}}$.

#### 2.1.2 Temporal 1D‐Conv

Inspired by Gehring et al. (2017) that CNNs have the superiority of fast training in sequential‐series analysis, we employ an entire convolutional structure on a temporal axis to capture sequential dynamic behaviours of EEG recordings. As shown in Figure 1a, a sequential convolutional layer contains a 1D convolution with a width $K_{t}$ kernel followed by ReLu (a rectified linear unit function) as a nonlinearity. For each node in graph G, its corresponding sequential convolution explores $K_{t}$ neighbours of input elements, leading to shortening the length of sequences by $K_{t}-1$ each time. Thus, an input of a sequential convolution for each node can be regarded as a length‐*M* sequence with $C_{i}$ channels as $Y\in R^{M\times C_{i}}$. The convolution kernel $\Gamma \in R^{K_{t}\times C_{i}\times C_{0}}$ is designed to map the input Y to a single output $P\in R^{\left(M-K_{t}+1\right)\times \left(C_{0}\right)}$. Similarly, the temporal convolution can be generalised to 3D variables by employing the same convolution kernel Γ to every channel node in G equally, noted as “Γ*TY” with $Y\in R^{M\times n\times C_{i}}$.

The input and output of ST‐Conv Blocks are all 3D tensors. For input $x^{l}\in R^{M\times n\times C_{l}}$ of block *l*, the output $x^{l+1}\in R^{\left(M-2\left(K_{t}-1\right)\right)\times n\times C^{l+1}}$ is computed by

(6) $$x^{l+1}=\Gamma_{1}^{l}*T\;\text{ReLU}\left(\Theta^{l}*G\left(\Gamma_{0}^{l}*T\;x^{l}\right)\right),$$

where $\Gamma_{0}^{l}$ and $\Gamma_{1}^{l}$ are the upper and lower temporal kernels within block *l*, respectively; $\Theta^{l}$ is the spectral kernel of a graph convolution; $\text{ReLU}\left(∙\right)$ denotes a rectified linear unit function. After stacking three ST‐Conv Blocks, the output features are fused as a flattened layer (Figure 1a). We can obtain a final output $Z\in R^{n\times c}$ from the fully connected layer and calculate the classification result by applying a sigmoid transformation as $\hat{\mathrm{Cl}}=\mathrm{sig}\left(\mathrm{Z\omega}+b\right)$, where $\omega \in R^{c}$ is a weight vector and b is a bias. We use the *binary cross‐entropy* loss to measure the classification performance.

All models were trained on the CPU of DELL DESKTOP‐D3UM3P9 with the Tensorflow platform in Microsoft Windows 10, and the optimiser used here is the Adam optimisation.

### 2.2 Adjacency matrix

The spatial information carried by EEG signals plays an important role in AD/HC classification (Babiloni et al., 2005; Sakkalis, 2011; Tafreshi et al., 2019; Van Mierlo et al., 2014). The adjacency matrix *W* $\in R^{n\times n}$ of each mini‐epoch, representing the spatial correlation along with the channel signals $X\in R^{n\times M}$ is one of the inputs of the ST‐GCN model shown in Figure 1a. In some studies on brain disorders based on GCN (Chen et al., 2020; Li, Liu, et al., 2021; Zeng et al., 2020; Zhang et al., 2021; Zhao et al., 2021), the relationship of different channels of EEG is short of effective prior guidance and the adjacency matrix cannot ensure the utilisation of the coupling information between each channel. To address these issues, we first apply functional connectivity, which has been proven to be useful in AD classification, to construct the adaptive adjacency matrix to extract spatial coupling features.

The raw EEG signals of each channel and the association of channels are modelled by a graph. The nodes of the graph denote the feature vector of EEG signals, which are the raw mini‐epoch EEG data. The adjacency matrix is constructed by Pearson correlation analysis and five functional connectivities of the mini‐epoch EEG channels without any preset threshold to avoid the potential risk caused by manual selection, such as in Chen et al. (2020), Zhang et al. (2021), and Zhao et al. (2021). Specifically, the (*i*,*j*)th entry of the adjacency matrix *W* denotes spatial coupling strength between the *i*th and *j*th channels. Thus, *W* indicates that all channels are interconnected with different weights. Pair‐wise correlation analysis among these 23 different channels is conducted using five functional connectivities and Pearson correlation for comparison, the calculation of which is presented below.

#### 2.2.1 Pearson correlation

The most well‐known functional connectivity measure is the correlation, also called the Pearson correlation coefficient. It calculates the instantaneous linear interdependency between two signals based on the amplitudes of the signals in the time domain and it ranges from −1 to 1. The Pearson correlation coefficient between signal X and Y can be defined as follows:

(7) $$\rho_{\mathrm{xy}}=\frac{E\left[\left(x-\mu_{x}\right)\left(y-\mu_{y}\right)\right]}{\sigma_{x}\sigma_{y}},$$

where E is the expected value, $\mu_{x}$ and $\mu_{y}$ are the mean values and $\sigma_{x}$ and $\sigma_{y}$ are the standard deviations of X and Y time series.

#### 2.2.2 Magnitude squared coherence

MSC is a linear method to estimate the interconnections between the PSD (power spectral density) of two signals in the frequency domain. The MSC of signals X and Y can be written as

(8) $${\mathrm{MSC}}_{\mathrm{xy}}\left(f\right)=C_{\mathrm{xy}}^{2}=\frac{S_{\mathrm{xy}}{\left(f\right)}^{2}}{\left|S_{\mathrm{xx}}\left(f\right)\right|\times \left|S_{\mathrm{yy}}\left(f\right)\right|},$$

where $S_{\mathrm{xx}}\left(f\right)$ and $S_{\mathrm{yy}}\left(f\right)$ are the PSD of signal X and Y, respectively, and $S_{\mathrm{xy}}\left(f\right)$ is the cross PSD at frequency f.

#### 2.2.3 Imaginary part of coherence

To avoid the volume conduction effects, instead of looking at the magnitude squared coherency, the imaginary part of the coherency is calculated by

(9) $${\mathrm{IC}}_{\mathrm{xy}}=RC_{\mathrm{xy}}+IC_{\mathrm{xy}}.$$

#### 2.2.4 Wavelet coherence

WC is generally acknowledged as a qualitative estimator that can represent the dynamic relations in the time–frequency domain between signals (Tafreshi et al., 2019). The wavelet transforms is defined as the convolution of the input x with a Wavelet family $\theta \left(u\right)$,

(10) $$W_{x}\left(t,f\right)=\int_{-\infty }^{+\infty }x\left(u\right)∙\theta_{t,f}^{*}\left(u\right)\mathrm{du}.$$

Given input signals x and y, wavelet cross‐spectrum around time t, and frequency f can be derived by the Wavelet transforms of x and y,

(11) $${\mathrm{CW}}_{\mathrm{xy}}\left(t,f\right)=\int_{t-\delta /2}^{t+\delta /2}W_{x}\left(\tau ,f\right)∙W_{y}^{*}\left(\tau ,f\right),$$

where * defines the complex conjugate and δ is assumed as a frequency‐depending time scalar. WC at the time t and frequency f is derived as

(12) $${\mathrm{WC}}_{\mathrm{xy}}\left(t,f\right)=\frac{\left|{\mathrm{CW}}_{\mathrm{xy}}\left(t,f\right)\right|}{{\left|{\mathrm{CW}}_{\mathrm{xx}}\left(t,f\right)\times {\mathrm{CW}}_{\mathrm{yy}}\left(t,f\right)\right|}^{1/2}}.$$

#### 2.2.5 Phase locking value

Phase synchronization assumes that two oscillation signals without amplitude synchronization can have phase synchronization. The PLV is high‐frequently utilised to obtain the strength of phase synchronisation (Van Mierlo et al., 2014). The instantaneous phase of a signal x is given by

(13) $$\emptyset_{x}\left(t\right)=\arctan\frac{\tilde{x}\left(t\right)}{x\left(t\right)},$$

where $\tilde{x}\left(t\right)$ is the Hilbert transform of $x\left(t\right)$, defined as

(14) $$\tilde{x}\left(t\right)=\frac{1}{\pi }\mathrm{PV}\int_{-\infty }^{+\infty }\frac{x\left(\tau \right)}{t-\tau }\mathrm{d\tau},$$

where PV refers to the Cauchy principal value. The PLV for two signals is then defined as

(15) $$\mathrm{PLV}=\left|\frac{1}{N}\sum_{j=0}^{N-1}e^{j\left(\emptyset_{x}\left(j∆t\right)-\emptyset_{y}\left(j∆t\right)\right)}\right|,$$

where ∆t defines the sampling period and N indicates the sample number of each signal. The range of PLV is between 0 and 1, where 0 shows a lack of synchronization and 1 indicates strict phase synchronization.

#### 2.2.6 Phase lag index

Similarly to the calculation of PLV, PLI captures the asymmetry of the distribution of phase differences between two signals and is calculated based on the relative phase difference between the two signals

(16) $$\mathrm{PLI}=\left|E\left[\mathrm{sign}\left(\emptyset_{x}\left(j∆t\right)-\emptyset_{y}\left(j∆t\right)\right)\right]\right|,$$

where $\emptyset_{x}\left(j∆t\right)-\emptyset_{y}\left(j∆t\right)$ is the phase difference between two signals, sign stands for signum function, E is the expected value, and || indicates the absolute value. PLI values range between 0 and 1, where 0 can indicate possibly no coupling and 1 refers to perfect phase locking.

### 2.3 Data set and preprocessing

All patients and healthy controls included in this work were recruited in the Sheffield Teaching Hospital memory clinic and all provided written informed consent. The EEG study underwent ethics approval by the Yorkshire and The Humber (Leeds West) Research Ethics Committee (reference number 14/YH/1070). AD patients had their diagnosis confirmed between 1 month and up to 2 years before recording their EEG while they had mild to moderate cognitive deficits, according to their Mini‐mental state examination. All AD subjects had brain MRI scans to eliminate other alternative causes of dementia. For the aged and gender‐matched HC cohort, normal MRI brain scans and cognitive assessments were required before their EEG recordings. Further details about recruitment, diagnostic criteria, and study design can be found in the previously published work (Blackburn et al., 2018).

All participants were younger than 70 years old, including 19 AD patients and 20 HC participants. EEG recordings were undertaken with an XLTEK 128‐channel headbox (Optima Medical Ltd.) and Ag/AgCL electrodes at a sampling frequency of 2 kHz by implementing a modified 10–10 overlapping a 10–20 international system of electrode placement, with a referential montage (linked earlobe reference). Thirty‐minute resting state (task‐free – participants were instructed to rest and refrain from thinking anything specific) EEG recordings were obtained from each participant including sustained periods of keeping their eyes closed (EC) alternating with periods during which they kept their eyes open (EO). The recordings obtained were subsequently reviewed by a neurophysiologist—on an XLTEK review station. For each participant, three 12‐s artefact‐free epochs of EC and EO were selected. To avoid volume conduction effects related to the common reference electrode, 23 bipolar derivations were created: F8–F4, F7–F3, F4–C4, F3–C3, F4–FZ, FZ–CZ, F3–FZ, T4–C4, T3–C3, C4–CZ, C3–CZ, CZ–PZ, C4–P4, C3–P3, T4–T6, T3–T5, P4–PZ, P3–PZ, T6–O2, T5–O1, P4–O2, P3–O1, and O1–O2.

For both EO and EC of 19 ADs and 20 HCs, there are three artefact‐free epochs, each of which lasts 12 s. We first applied the Butterworth filter for every epoch to subsample the EEG signals at 100 Hz and obtained the corresponding six bands (Delta, Theta, Alpha, Beta, Gamma, and Full band of 0–48 Hz). Then, data segmentation is utilised to obtain 5472 mini‐epochs for ADs and 5760 mini‐epochs for HCs with a window size of 25 data points without overlapping. For each mini‐epoch signal, six functional connectivities (PC, MSC, IPC, WC, PLV, and PLI) were calculated to obtain the corresponding adjacency matrices. Finally, for each measure of EC or EO, 3744 samples (1824 for ADs and 1920 for HCs) were selected randomly covering two‐thirds of each 12 s epoch and split for 10‐folder cross‐validation to generate the training data set and validation data set. The remaining 1872 samples (912 for ADs and 960 for HCs) were considered as the testing data set. The purpose of the validation data set during training is to obtain the model loss on the validation data set after the optimization of each epoch. If the loss of the validation data set is decreasing, we save the optimised model until the end of all training epochs, which can prevent the model from overfitting.

## 3 RESULTS

### 3.1 Adjacency matrix from different functional connectivities

**FIGURE 2:**
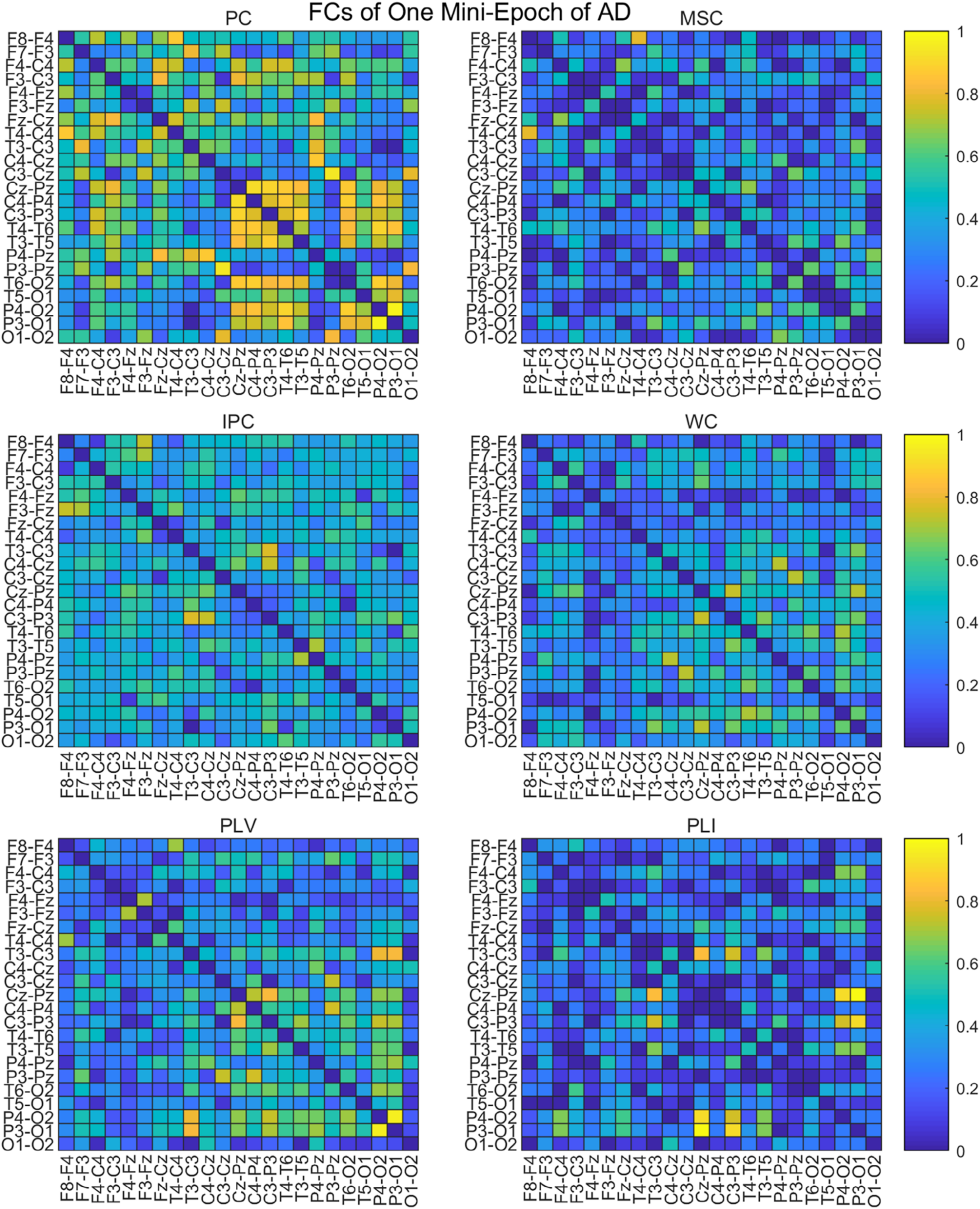
The adjacency matrices of one mini‐epoch of AD from six FC methods

**FIGURE 3:**
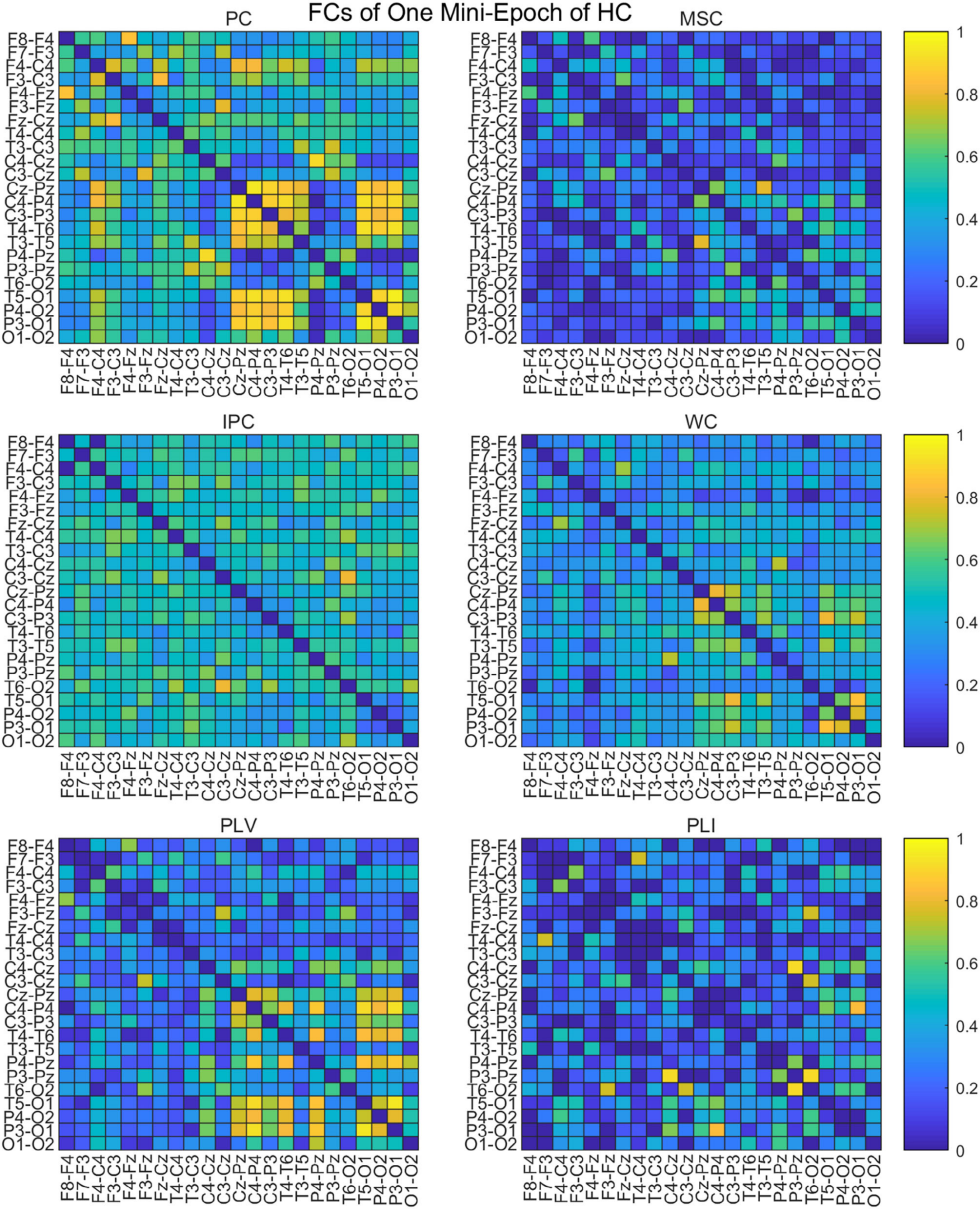
The adjacency matrices of one mini‐epoch of HC from six FC methods

To compare the adjacency matrices calculated by six functional connectivities, we take the Full band of one mini‐epoch of AD (Figure 2) and HC (Figure 3) as an example to see their difference. For the mini‐epoch signal of both AD and HC, PC generates higher coupling between channels than the five functional connectivity methods. The adjacency matrix calculated by MSC is relatively lower than the others and WC and PLV show similar coupling distribution with PC but with overall lower strength. IPC produces the most uniform connection distribution. PLI produces the sparsest connection distribution, and there are some strong connection points close to 1. Therefore, adjacency matrices from the six calculation methods have differences in the coupling analysis between EEG channels, which may have an impact on the classification performance of the ST‐GCN method.

### 3.2 Overall classification performance

We conducted a direct comparison of classifying AD and HC participants using the extracted six adjacency matrices by training the ST‐GCN and T‐CNN methods on the EO and EC states and six frequency bands independently. Tables 1 and 2 show the corresponding testing accuracies from ST‐GCN and T‐CNN models, respectively. For both methods, the classification accuracy of all frequency bands and adjacency matrix calculation methods in the EC state is higher than the corresponding EO state, which is consistent with previous research results (Barry et al., 2007; Wan et al., 2019). For different frequency bands, when the 1–48 Hz full‐band signal and the corresponding adjacency matrix are used as the input, the mean classification accuracies are about 91.1% by ST‐GCN and 88.0% by T‐CNN, respectively, which are higher than all sub‐bands data. For ST‐GCN, the mean classification accuracy of Beta band data is the highest (about 82.3% for EC and 77.7% for EO), and the mean classification accuracy of Delta band data is the lowest (about 73.1% for EC and 71.0% for EO). For T‐CCN, the mean classification accuracy of Alpha band data is the highest (about 76.8% for EC and EO), and the mean classification accuracy of Delta band data is the lowest (about 68.6% for EC and EO). Overall, ST‐GCN outperforms T‐CNN on both eye states and almost all sub‐band data, which indicates that spatial topology constraints can indeed mine EEG features, resulting in improved classification accuracy.

**TABLE 1:** Classification accuracy for HC and AD in EC and EO states within six bands by ST‐GCN with different adjacency matrices

Eye states	FC	Delta	Theta	Alpha	Beta	Gamma	Full
EC	PC	72.1	78.2	78.3	82.5	81.2	90.3
	MSC	72.2	78.1	78.9	81.9	80.8	90.2
	IPC	73.8	78.4	80.2	82.3	82.1	90.7
	WC	73.9	78.7	81.0	83.2	82.5	92.3
	PLV	73.0	78.6	79.8	82	82.2	91.1
	PLI	73.9	78.2	79.6	82.3	81.1	92.1
EO	PC	70.9	75.6	76.8	77.4	75.5	88.1
	MSC	71.3	75.7	75.4	77.8	75.5	88.3
	IPC	71.0	76.0	77.4	78.1	75.6	88.7
	WC	71.2	76.8	78.2	78.5	76	89.4
	PLV	71.0	75.8	76.0	78.1	75.9	87.2
	PLI	70.3	75.0	75.6	76.5	75.8	87.9

Abbreviations: IPC, imaginary part of coherence; MSC, magnitude squared coherence; PC, Pearson correlation; PLI, phase lag index; PLV, phase locking value; WC, wavelet coherence.

**TABLE 2:** Classification accuracy for HC and AD in EC and EO states within six bands by T‐CCN with different adjacency matrices

Eye states	FC	Delta	Theta	Alpha	Beta	Gamma	Full
EC	PC	69.0	76.7	77.3	80.3	75.9	88.1
	MSC	70.4	76.1	74.0	80.7	74.8	86.8
	IPC	69.4	77.8	77.1	78.2	77.5	88.8
	WC	71.0	78.0	76.7	80.3	80.5	89.0
	PLV	71.4	78.2	76.9	77.9	77.4	88.1
	PLI	68.5	76.6	78.7	80.6	75.5	87.4
EO	PC	67.9	75.3	77.2	71.3	74.3	85.6
	MSC	67.0	74.8	76.9	75.0	69.8	85.9
	IPC	65.0	76.1	76.5	72.9	74.1	86.4
	WC	70.1	75.9	77.9	76.2	73.7	86.3
	PLV	65.8	74.5	75.9	71.5	73.5	85.0
	PLI	67.3	74.9	76.9	71.6	71.9	85.7

Abbreviations: IPC, imaginary part of coherence; MSC, magnitude squared coherence; PC, Pearson correlation; PLI, phase lag index; PLV, phase locking value; WC, wavelet coherence.

It is worth noting that for different adjacency matrix calculation methods, the classification accuracy of the WC method is better than all other methods, which proves the effectiveness of time–frequency analysis for extracting coupling features. For ST‐GCN, the overall accuracy of the five functional connection methods is higher than that of the PC method, indicating that the usage of functional connectivity as the adjacency matrix can improve the performance of the ST‐GCN model to a certain extent.

**FIGURE 4:**
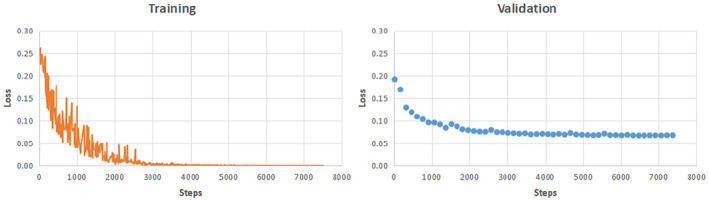
Training and convergence process for Full band data with WC connectivity by the ST‐GCN model

The training and convergence process for Full band data with WC connectivity by the proposed ST‐GCN model is shown in Figure 4.

### 3.3 Wavelet coherence as adjacency matrix

**FIGURE 5:**
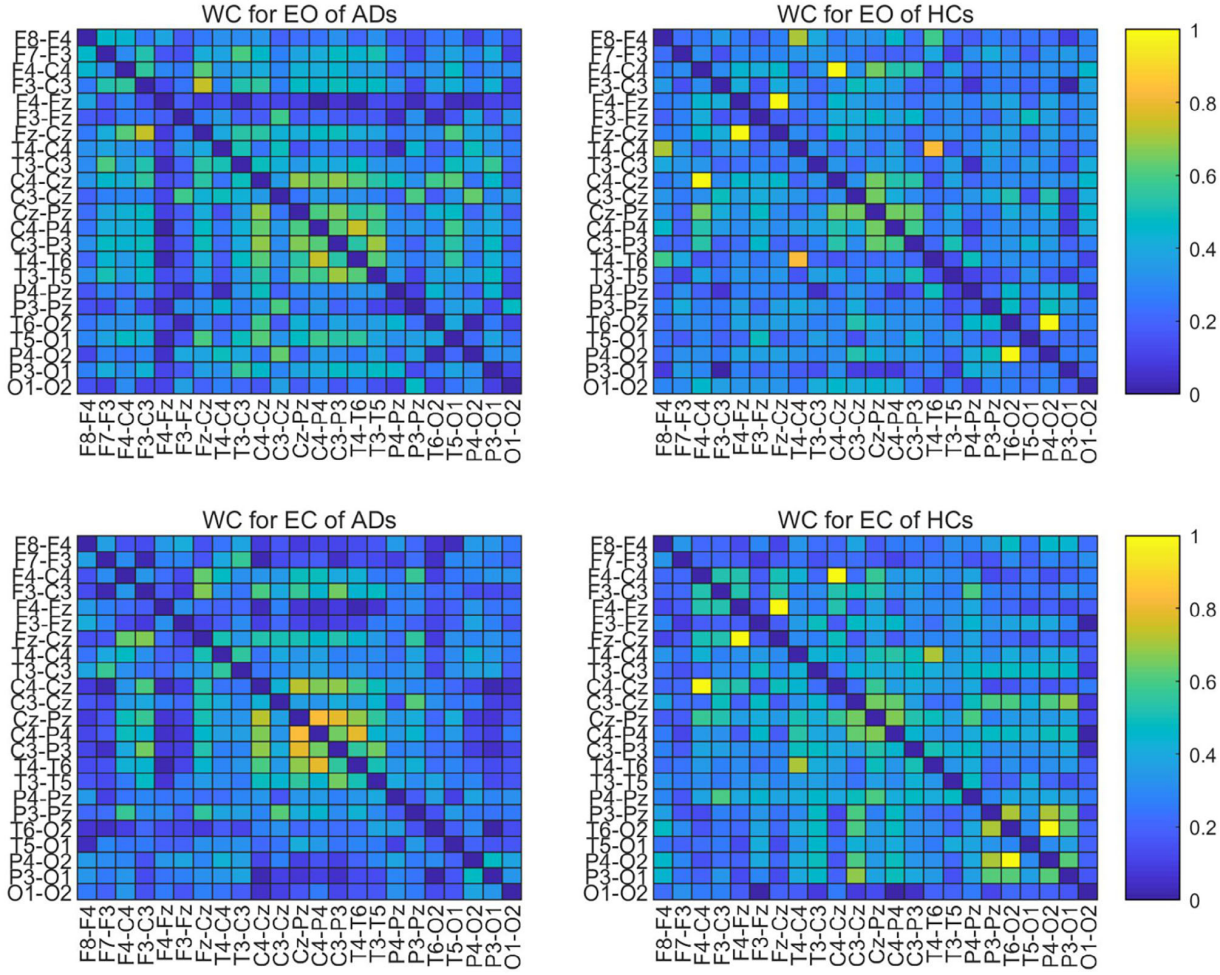
The averaged WC adjacency matrices of ADs and HCs

To further explore the effectiveness of WC which has the best classification performance as the adjacency matrix, we conduct a statistical analysis of the WCs of all AD and HC. By averaging the WC adjacency matrices of the full band of all EO and EC mini‐epochs for AD and HC, respectively, as shown in Figure 5, we can analyse their statistical characteristics. For ADs, the EC state has a slightly higher coupling strength than EO in the temporo‐occipital area (the middle part of the adjacency matrix), while for HCs, the EC state has a bit higher overall coupling strength than EO. For both EO and EC, the strongest interchannel correlation of AD is less than 0.8 (the middle part of the left two images of Figure 5 – temporo‐occipital area), while HC has some interchannel correlations close to 1 (the upper‐left frontocentral and bottom‐right posterior area of the right two images of Figure 5). For both EO and EC, the connectivity between ADs channels is lower than that of HCs in the bottom‐right corner (posterior area).

**FIGURE 6:**
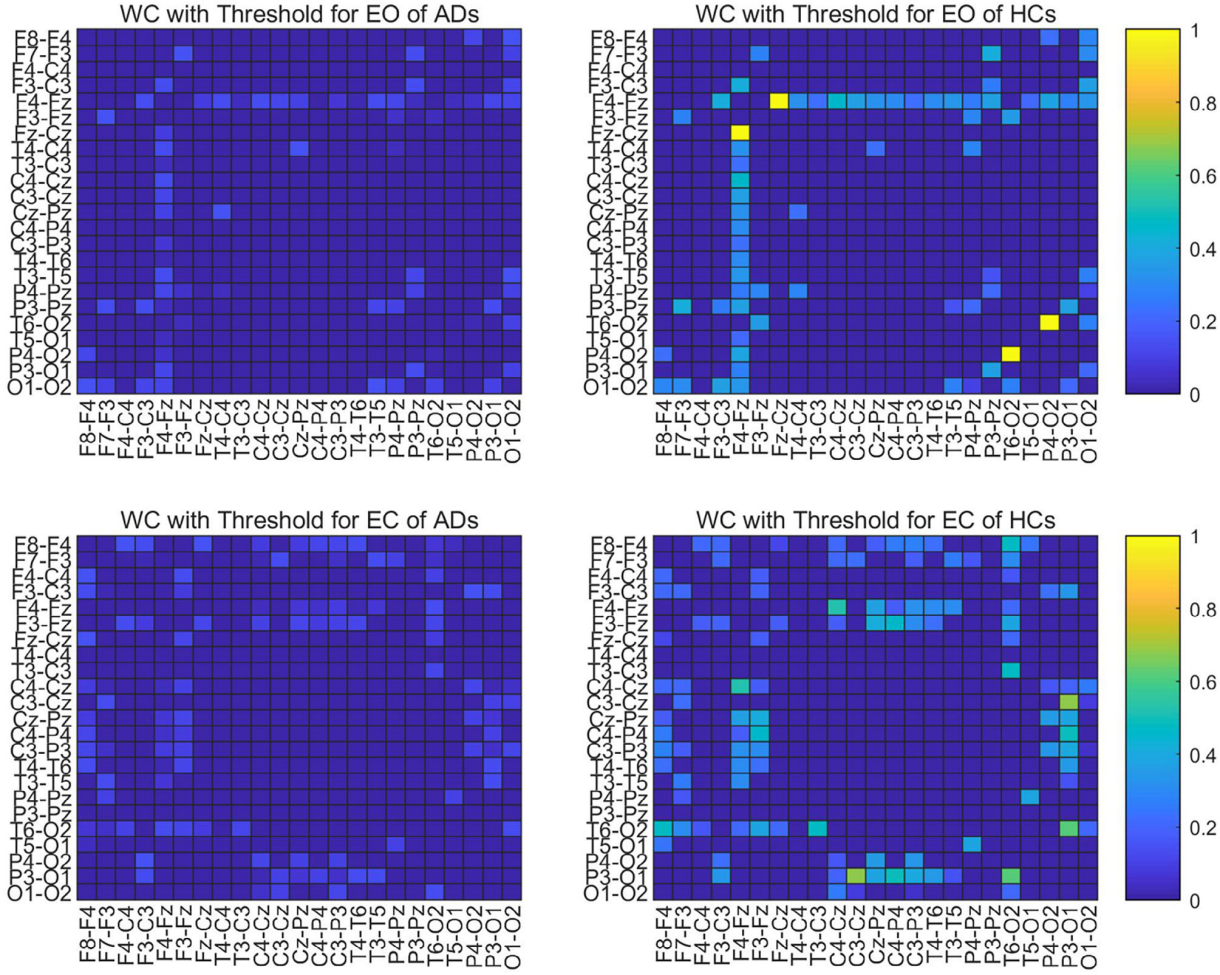
Threshold of averaged WC adjacency matrices of ADs and HCs

**FIGURE 7:**
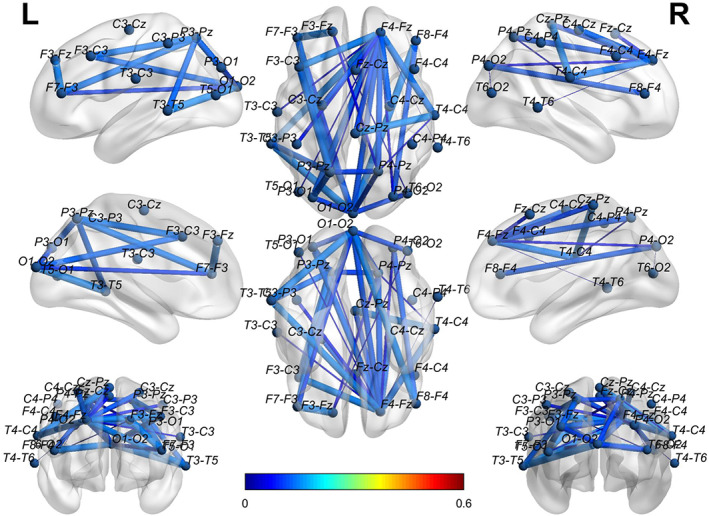
The 3D brain mapping of averaged WC adjacency matrices for EO state of ADs

**FIGURE 8:**
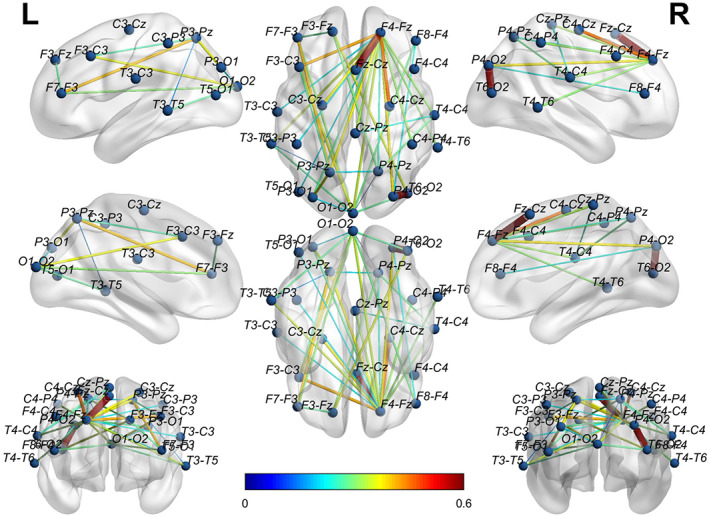
The 3D brain mapping of averaged WC adjacency matrices for EO state of HCs

**FIGURE 9:**
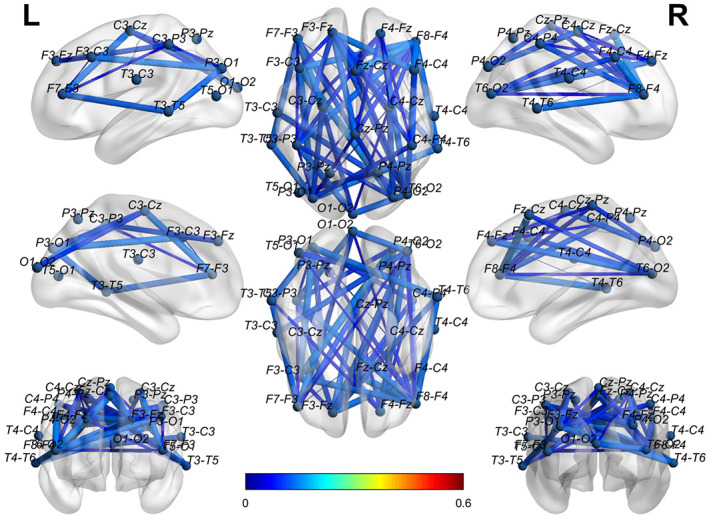
The 3D brain mapping of averaged WC adjacency matrices for EC state of ADs

**FIGURE 10:**
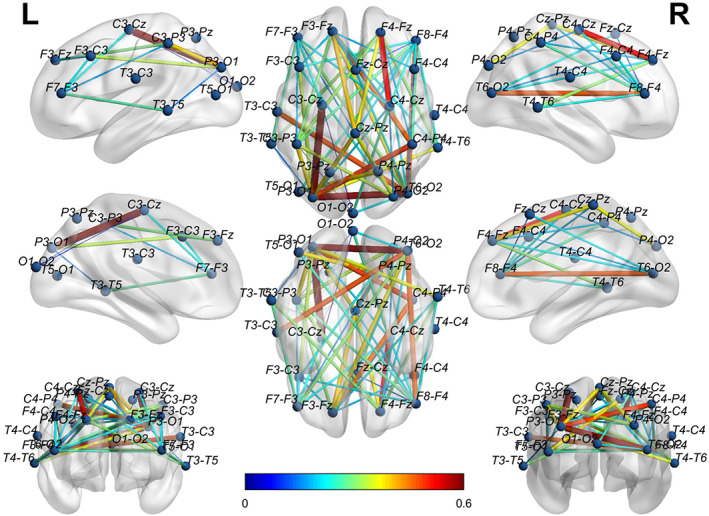
The 3D brain mapping of averaged WC adjacency matrices for EC state of HCs

To visually show the connectivity between the channels in the bottom‐left corner for both EO and EC states, we thresholded the left two AD adjacency matrices in Figure 5 to screen out the channels with connectivity below 0.15. Figure 6 reports the corresponding cross‐channel indexes for HCs. Through the software BrainNet, the 3D connectivity distributions in the brain of ADs and HCs are visually displayed for EO (Figures 7 and 8) and EC (Figures 9 and 10), respectively. Comparing Figures 7 and 8 for EO, the connectivities of HCs between the channels at the front and back parts of the brain are much higher than those of ADs. P4‐O2 to T6‐O2 and F4‐Fz to Fz‐Cz have the highest coupling strengths (close to 0.6) for channel pairs of HCs, and the corresponding coupling strengths of ADs are close to 0. Comparing Figures 9 and 10 for EC, the connectivity of HCs between the channels at the back of the brain is much higher than that of ADs. C3‐Cz to P3‐O1 and P3‐O1 to T6‐O2 have the highest coupling strengths (close to 0.6) for channel pairs of HCs, and the corresponding coupling strengths of ADs are as low as 0.1.

## 4 CONCLUSIONS

We proposed a Spatial–temporal Graph Convolutional Network (ST‐GCN) for classifying Alzheimer's disease and Healthy Controls groups by jointly leveraging cross‐channel topological association features and channel‐specific temporal features of EEG recordings. Different from the currently leading GCN applications for diagnosing brain disorders, this method utilises brain functional connectivity methods for exploring the complex interactive information between EEG channels as well as the single‐channel‐based dynamic information. The main goal of this work was to determine whether the cross‐channel topologically associated features constrained by the functional connectivity can reveal more hidden information in data and extend the applicability of the GCN‐based algorithm. For the clinical AD and HC EEG recordings, ST‐GCN has exhibited superior performance in achieving the highest classification accuracy with wavelet coherence as the adjacency matrix. For the tested data set, the overall classification accuracy of ST‐GCN is higher than the classical T‐CNN method on both eye states and different frequency bands, which suggests that spatial topology constraints can indeed mine brainwave features and thereby improve the classification accuracy. Different from existing studies for AD diagnosis that are based on either topological brain function characteristics of EEG (Fan et al., 2018; Jalili, 2017; Tait et al., 2020; Tylová et al., 2018; Zhao et al., 2019) or temporal dynamic features (Bi & Wang, 2019; Deepthi et al., 2020; Huggins et al., 2021; Ieracitano et al., 2019; Li, Wang, et al., 2021), the proposed ST‐GCN considers both the adjacency matrix of functional connectivity from multiple EEG channels and corresponding dynamics of signal EEG channel simultaneously. It has the potential to pick up the anomaly of AD not only in the frequency response of local areas but also in the functional connectivity across different regions. Furthermore, the visualisation of wavelet coherence adjacency matrices increases the transparency of this solution by providing evidence of brain anomaly in terms of functional connectivity. This investigation is important as it will increase the trust in the developed AI‐based solution. This algorithm lays a potentially effective strategy for the applications of other brain disorders.

In the present study, due to the limited number of subjects in the data set, the Leave‐One‐Subject‐Out or cross‐subject validation is not discussed to avoid biased conclusions caused by data insufficiency. The accuracy of a hand‐out cross‐subject validation by ST‐GCN and T‐CNN can be found in Tables S1 and S2, Supporting Information. In this case, 65% of the subjects were used for training and the remaining 35% were used for testing. Although the overall accuracy is dropped significantly for both methods, the proposed ST‐GCN still outperforms T‐CNN.

To reduce volume conduction effects from a common reference, bipolar derivations were used to assess the degree of differences between various pairs of electrodes for two different cohorts of subjects. With this approach—the use of bipolar pairs of electrodes—the effects of volume conduction are reduced but not eliminated. We recognise that this work is based on a sensor level scalp EEG analysis, and we do not claim to be able to precisely localise the spatial characteristics underpinning the EEG sensor findings.

## Supporting information


**Table S1** ST‐GCN Model performance in a hand‐out validation, where 12 ADs and 13 HCs were used for training and validation and the remaining 7 ADs and 7 HCs were used for testing. Overall, the classification accuracy of the full‐band data is still the best, with an average of 70.5% for EC and 68.9% for EO.
**Table S2** T‐CCN Model performance in a hand‐out validation, where 12 ADs and 13 HCs were used for training and validation and the remaining 7 ADs and 7 HCs were used for testing. Overall, the classification accuracy of the full‐band data is lower than the ST‐GCN model.

## Data Availability

Data sharing is not applicable to this article as no new data were created or analyzed in this study.
