# Supplementary material for: Spatial–temporal graph convolutional network for Alzheimer classification based on brain functional connectivity imaging of electroencephalogram
Source: Hum Brain Mapp. 2022 Jun 25;43(17):5194–209. doi: 10.1002/hbm.25994 (PMC9812255; doi:10.1002/hbm.25994)
Supplement: Supplementary file 1 — Table S1 ST‐GCN Model performance in a hand‐out validation, where 12 ADs and 13 HCs were used for training and validation and the remaining 7 ADs and 7 HCs were used for testing. Overall, the classification accuracy of the full‐band data is still the best, with an average of 70.5% for EC and 68.9% for EO. Table S2 T‐CCN Model performance in a hand‐out validation, where 12 ADs and 13 HCs were used for training and validation and the remaining 7 ADs and 7 HCs were used for testing. Overall, the classification accuracy of the full‐band data is lower than the ST‐GCN model. [file HBM-43-5194-s001.docx]

**Table S1 ST-GCN Model performance in a hand-out validation, where 12 ADs and 13 HCs were used for training and validation and the remaining 7 ADs and 7 HCs were used for testing. Overall, the classification accuracy of the full-band data is still the best, with an average of 70.5% for EC and 68.9% for EO.**

|  | **SUBJECT INDEPENDENT** | | | | | |  |  |
| --- | --- | --- | --- | --- | --- | --- | --- | --- |
|  |  |  |  |  |  |  |  |  |
| **Eye States** | **FC** | **Delta** | **Theta** | **Alpha** | **Beta** | **Gamma** | **Full** |  |
| **EC** | **PC** | 56.9 | 67.1 | 65.6 | 58.0 | 45.8 | 70.9 |  |
|  | **MSC** | 56.6 | 67.7 | 65.1 | 59.1 | 51.7 | 66.7 |  |
|  | **IPC** | 56.9 | 67.9 | 63.9 | 58.1 | 49.0 | 70.6 |  |
|  | **WC** | 58.5 | 69.8 | 67.9 | 59.3 | 49.6 | 72.1 |  |
|  | **PLV** | 51.0 | 68.1 | 66.3 | 57.9 | 48.6 | 71.1 |  |
|  | **PLI** | 56.1 | 69.3 | 64.3 | 55.7 | 46.8 | 71.5 |  |
| **EO** | **PC** | 53.0 | 68.8 | 63.8 | 61.3 | 54.7 | 68.2 |  |
|  | **MSC** | 57.7 | 67.2 | 63.1 | 56.0 | 52.3 | 67.3 |  |
|  | **IPC** | 54.7 | 68.6 | 65.3 | 55.3 | 56.9 | 69.6 |  |
|  | **WC** | 57.1 | 69.1 | 65.4 | 61.0 | 57.1 | 70.2 |  |
|  | **PLV** | 55.7 | 66.6 | 61.9 | 56.1 | 55.4 | 69.6 |  |
|  | **PLI** | 55.3 | 68.1 | 63.5 | 54.9 | 52.8 | 68.5 |  |
| Abbreviations: PC, Pearson Correlation; MSC, Magnitude Squared Coherence; IPC, Imaginary Part of Coherence; WC, Wavelet Coherence; PLV, Phase Locking Value; PLI, Phase Lag Index. | | | | | | | |  |
|  |  |  |  |  |  |  |  |  |
|  |  |  |  |  |  |  |  |  |

**Table S2 T-CCN Model performance in a hand-out validation, where 12 ADs and 13 HCs were used for training and validation and the remaining 7 ADs and 7 HCs were used for testing. Overall, the classification accuracy of the full-band data is lower than the ST-GCN model.**

|  | **SUBJECT INDEPENDENT** | | | | | |  |  |
| --- | --- | --- | --- | --- | --- | --- | --- | --- |
|  |  |  |  |  |  |  |  |  |
| **Eye States** | **FC** | **Delta** | **Theta** | **Alpha** | **Beta** | **Gamma** | **Full** |  |
| **EC** | **PC** | 52.9 | 70.6 | 62.6 | 54.6 | 50.3 | 66.4 |  |
|  | **MSC** | 58.2 | 67.2 | 62.2 | 56.5 | 47.6 | 64.4 |  |
|  | **IPC** | 52.8 | 66.5 | 63.3 | 60.1 | 44.9 | 66.5 |  |
|  | **WC** | 55.9 | 64.8 | 62.4 | 59.7 | 50.6 | 65.5 |  |
|  | **PLV** | 54.1 | 69.5 | 62.8 | 59.6 | 57.8 | 67.6 |  |
|  | **PLI** | 56.8 | 67.5 | 61.8 | 55.5 | 53.3 | 66.1 |  |
| **EO** | **PC** | 50.9 | 69.2 | 64.4 | 60.9 | 55.7 | 66.5 |  |
|  | **MSC** | 48.7 | 68.3 | 64.8 | 54.9 | 54.7 | 66.1 |  |
|  | **IPC** | 53.7 | 65.3 | 66.3 | 57.8 | 55.0 | 64.4 |  |
|  | **WC** | 50.8 | 69.2 | 66.2 | 58.4 | 56.7 | 69.5 |  |
|  | **PLV** | 52.5 | 66.8 | 60.5 | 57.7 | 52.7 | 66.7 |  |
|  | **PLI** | 49.8 | 68.2 | 64.3 | 57.7 | 55.0 | 61.7 |  |
| Abbreviations: PC, Pearson Correlation; MSC, Magnitude Squared Coherence; IPC, Imaginary Part of Coherence; WC, Wavelet Coherence; PLV, Phase Locking Value; PLI, Phase Lag Index. | | | | | | | |  |
|  |  |  |  |  |  |  |  |  |
|  |  |  |  |  |  |  |  |  |
